# Characterization of disease flares and impact of mepolizumab in patients with hypereosinophilic syndrome

**DOI:** 10.3389/fimmu.2022.935996

**Published:** 2022-08-26

**Authors:** Fabrizio Pane, Guillaume Lefevre, Namhee Kwon, Jane H. Bentley, Steven W. Yancey, Jonathan Steinfeld

**Affiliations:** ^1^ Division of Hematology, Department of Clinical Medicine and Surgery, Federico II University, Naples, Italy; ^2^ Université de Lille, CHU Lille, Institut d’Immunologie, Centre de Référence National des Syndromes Hyperéosinophiliques, Institute for Translational Research in Inflammation Infinite-U1286, Inserm, Lille, France; ^3^ Respiratory Research and Development, GSK, Brentford, United Kingdom; ^4^ Clinical Statistics, GSK, Brentford, United Kingdom; ^5^ Respiratory Therapeutic Area, GSK, Durham, NC, United States; ^6^ Respiratory Research and Development, GSK, Collegeville, PA, United States

**Keywords:** antibody, anti-interleukin-5 therapy, hypereosinophilic syndrome, mepolizumab, uncontrolled disease

## Abstract

In patients with hypereosinophilic syndrome (HES), mepolizumab reduces the incidence of HES-related clinical signs and symptoms (flares). However, reports characterizing flare manifestations are limited. The double-blind, parallel-group 200622 trial (NCT02836496) enrolled patients ≥12 years old with HES for ≥6 months, ≥2 flares in the previous year, and screening blood eosinophil count ≥1000 cells/μL. Patients maintained ≥4 weeks stable HES therapy, before randomization (1:1) to 4-weekly subcutaneous mepolizumab (300 mg) or placebo, plus baseline HES therapy, for 32 weeks. This *post hoc* analysis investigated flare manifestations and duration by re-examining the Core Assessments form and narrative recorded for each flare during the study. Flare symptoms were retrospectively categorized into constitutional, dermatological, respiratory, nasal, gastrointestinal, neurologic and other. The most frequently reported flare symptoms were constitutional (94% of flares), dermatological (82% of flares) and respiratory (72% of flares); flares reported in patients receiving mepolizumab compared with placebo were generally similar in terms of the frequency of symptoms reported. Mepolizumab was associated with a shorter median (range) duration of flares (10.0 [4, 126] days) versus placebo (26.0 [1, 154] days). In patients with HES, flares were associated with symptoms linked to multiple organ systems highlighting the challenges faced for treating flares.

**Clinical Trial Registration:**

https://clinicaltrials.gov/ct2/show/NCT02836496, identifier NCT02836496.

## Introduction

Hypereosinophilic syndrome (HES) is a rare disorder characterized by prolonged, elevated eosinophil counts in the peripheral blood and/or tissues and eosinophil-mediated organ damage and/or dysfunction that is not secondary to classical causes of hypereosinophilia (1, 2). The clinical presentation of HES is heterogenous as any tissue or organ system can be affected. The most common symptoms reported relate to the skin, lung, and gastrointestinal system (1, 3–5), and patients may experience multiple symptoms and multiple organ system involvement over the course of their disease (3, 6, 7). HES can be subcategorized based on clinical, laboratory, and molecular features, and the subcategories include idiopathic, lymphocytic, and myeloproliferative (1, 2, 8).

The goals of disease management for patients with HES are to control their symptoms and mitigate tissue damage (8). Periods of worsening in HES-related clinical signs and symptoms, termed flares, are not only associated with significant morbidity and impact patients’ health-related quality of life, but can also be life-threatening (9–12). Flares frequently necessitate treatment and the current standard of care includes recurrent and sustained use of oral corticosteroids (OCS), in conjunction with immunosuppressant and/or cytotoxic therapies (IS/CT) (9, 13, 14). Studies have shown that these treatments reduce elevated eosinophil counts; however, alternative treatment options may be desired owing to their variable clinical efficacy and substantial concomitant side effects (1, 3, 13, 15–17).

The humanized, monoclonal anti-interleukin (IL)-5 antibody, mepolizumab, binds to IL-5, a key regulator of eosinophil proliferation, activation, and survival, and inhibits its interaction with the IL-5 receptor (18). Mepolizumab is approved for the treatment of severe eosinophilic asthma and eosinophilic granulomatosis with polyangiitis in multiple regions, and more recently, for HES in the USA, EU and Brazil and for chronic rhinosinusitis with nasal polyps in the USA and EU (19–21). The approval of mepolizumab for HES was based on the results of the double-blind, Phase III 200622 study (NCT02836496), in which the efficacy of mepolizumab, in addition to standard of care therapy, was assessed in patients with HES. In particular, mepolizumab was found to be associated with a 50% reduction in the proportion of patients experiencing at least one flare during the 32-week study and a 66% reduction in the annualized flare rate versus placebo, with no new safety signals identified (22). The objective of this *post hoc* analysis of data from the 200622 study was to further characterize flare manifestations and to assess the impact of mepolizumab on flare duration.

## Materials and methods

### Study design and patients

This was a *post hoc* analysis of data from the randomized, placebo-controlled, double-blind, parallel-group, multicenter, Phase III trial (NCT02836496; study 200622); full details of the this study have been reported previously (22). Briefly, patients were randomized (1:1) to receive mepolizumab 300 mg subcutaneously (SC) or placebo every 4 weeks for 32 weeks, in addition to their existing HES therapy.

Key patient eligibility criteria included patients ≥12 years of age at screening with a diagnosis of HES (excluding *FIP1L1-PDGFRA*-positive HES) for ≥6 months before screening. HES diagnosis was based on organ system involvement and/or dysfunction that could be directly related to a blood eosinophil count >1500 cells/μL on ≥2 occasions, and/or tissue eosinophilia, without a discernible secondary cause. Patients were receiving stable HES therapy for ≥4 weeks before the baseline visit (first administration of mepolizumab or placebo), had experienced ≥2 flares within the past 12 months and had a baseline blood eosinophil count ≥1000 cells/μL at screening. Baseline HES therapy could include OCS with or without IS/CT.

The trial was conducted in accordance with the ethical principles of the Declaration of Helsinki, the International Conference on Harmonisation Good Clinical Practice guidelines, and applicable country-specific regulatory requirements. The local institutional review board or ethics committee at each study center oversaw trial conduct and documentation (see list of approving ethics committees for the HES 200622 study section for further details). All patients provided written informed consent.

### *Post hoc* analysis endpoints

In the 200622 study, flares were defined as a) a HES-related clinical manifestation (based on a physician-documented change in clinical signs or symptoms) that required either an increased dose of maintenance OCS ≥10 mg prednisone equivalent/day for 5 days or an increase in/addition of IS, or b) receipt of ≥2 courses of blinded OCS during the treatment period (22). For definition a) flares, investigators evaluated patient’s clinical manifestations at each clinic visit using the HES Core Assessments form (further details in **Supplementary File 1** and **Supplementary Table 1**) and determined whether worsening of signs/symptoms supported an increase in HES therapy; clinical judgement determined whether the patient was experiencing a flare. In addition, investigators reported a narrative for each flare. Definition b) flares were determined from the clinical database at the end of the study.

In this *post hoc* analysis, a clinical review of flare characteristics for HES flares meeting flare definition a) was performed based on the information in the HES Core Assessments form and the flare narrative recorded during the study. The start date for a HES flare meeting definition a) was determined as the date of therapy escalation confirmed by the investigator attributable to a HES-related clinical manifestation, to ensure clinically significant worsening of symptoms, i.e. those requiring therapy escalation, were captured. The end date for a HES flare was determined by the investigator as the date the flare was resolved. Based on this review, flare symptoms were retrospectively categorized into constitutional, dermatological, respiratory, nasal (ear, nose, throat), gastrointestinal, neurologic, and other categories; a cardiovascular category was present in the HES Core Assessments form; however, no patients in this study had cardiovascular symptoms associated with HES flares. A sub-categorization for the individual symptom within each of the seven main symptoms categories was also assigned (broadly based on the HES Core Assessments form, **Supplementary Table 1**). Each HES flare could be categorized with more than one individual symptom and across several symptom categories, as appropriate, based on the review.

### Statistical analysis

Analyses were performed *post hoc*. Only flares meeting definition a) were included in this analysis as they were dependent on clinical signs and symptoms. Flares meeting protocol definition a) with less than 14 days between the flare start date and the end date of a previous flare (meeting either flare definition) were counted as a single flare. For flares meeting both definition a) and b), total flare duration was assigned based on the start and end dates recorded by the investigator i.e., the duration where the flare met definition a). In flares with a missing end date, the duration of flare was calculated up to date of study withdrawal.

The primary analysis population was the intent-to-treat (ITT) population, defined as randomized patients who received ≥1 dose of study treatment. Medical conditions and baseline therapy at screening were summarized in the total study population and in subgroups according to the clinical duration of disease (≤5, >5 – ≤ 10 >10 years since diagnosis). The proportion of flares in each of the 7 symptom categories (constitutional, dermatological, respiratory, nasal, gastrointestinal, neurologic, and other) was summarized for the total population, by treatment group, and in subgroups according to baseline blood eosinophil count (<1500 cells/µL, ≥1500–<2500 cells/µL, ≥2500 cells/µL), baseline HES therapy (IS/CT [± OCS], OCS no IS/CT, no IS/CT/OCS) and clinical duration of disease (≤5 years, 5–≤10 years, >10 years since diagnosis).

The frequency of individual symptoms within each category was also described for the total study population. Flare duration up to Week 32 was summarized for the total study population. The adjusted mean rate/year of HES flares meeting definition a) was determined using a negative binomial generalized linear model including baseline OCS dose, treatment, region and observed time (as an offset variable). All analyses were performed using SAS software, version 9.4 (SAS Institute Inc., Cary, NC, USA).

## Results

### Patient demographics and clinical characteristics

In total, 108 patients (mepolizumab, n=54; placebo, n=54) were included in the ITT population of the 200622 study; patient demographics and clinical characteristics at baseline for these patients have been reported previously (22). Most patients had ≥1 medical condition at screening (81%), the most common being metabolism and nutritional (35%), respiratory, thoracic and mediastinal (32%) and nervous system (31%) disorders (**Table 1**). Medical conditions at screening occurred more frequently in the subgroup with HES duration >10 years than in those with HES duration ≤5 years (94% and 76%, respectively, **Table 1**).

**Table 1:** Medical conditions and baseline therapy by duration of HES.

	Total (n = 108)	HES duration (years)			
		≤5 (n = 70)	>5–≤10 (n = 20)	>10 (n = 18)	
**Current medical condition, n (%)**		** **	** **	** **	
Any	87 (81)	53 (76)	17 (85)	17 (94)	
Metabolism and nutrition disorder	38 (35)	20 (29)	9 (45)	9 (50)	
Weight gain	19 (18)	9 (13)	6 (30)	4 (22)	
Osteoporosis	13 (12)	6 (9)	2 (10)	5 (28)	
Hypercholesterolemia	12 (11)	8 (11)	1 (5)	3 (17)	
Diabetes mellitus	6 (6)	2 (3)	1 (5)	3 (17)	
Respiratory, thoracic, and mediastinal disorder	35 (32)	24 (34)	4 (20)	7 (39)	
Allergic rhinitis or hay fever	27 (25)	18 (26)	4 (20)	5 (28)	
Nasal polyposis	14 (13)	11 (16)	1 (5)	2 (11)	
Nervous system disorder	34 (31)	21 (30)	8 (40)	5 (28)	
Endocrine disorder	25 (23)	9 (13)	8 (40)	8 (44)	
Other disorder	25 (23)	13 (19)	7 (35)	5 (28)	
Vascular disorder	24 (22)	16 (23)	5 (25)	3 (17)	
Infection and infestation	16 (15)	11 (16)	3 (15)	2 (11)	
Cardiac disorder	13 (12)	8 (11)	4 (20)	1 (6)	
Eye disorder	6 (6)	2 (3)	3 (15)	1 (6)	
Hepatobiliary disorder	2 (2)	1 (1)	1 (5)	0	
**Baseline HES therapy, n (%)**					
Any	99 (92)	66 (94)	18 (90)	15 (83)	
Prednisone equivalent OCS daily dose					
0 mg	30 (28)	21 (30)	4 (20)	5 (28)	
>0–≤20 mg	72 (67)	45 (64)	15 (75)	12 (67)	
>20 mg	6 (6)	4 (6)	1 (5)	1 (6)	
IS*	23 (21)	14 (20)	8 (40)	1 (6)	
Other^†^	41 (38)	27 (39)	9 (45)	5 (28)	
No OCS/IS	25 (23)	16 (23)	4 (20)	5 (28)	
**Prednisone equivalent OCS daily dose (mg), median (range)**	5.6 (0, 50)	6.3 (0, 50)	7.5 (0, 25)	5.0 (0, 50)	

*Including, but not limited to, hydroxycarbamide, ciclosporin, imatinib, methotrexate, tacrolimus, azathioprine; ^†^including, but not limited to, beclometasone dipropionate, formoterol fumarate, omeprazole, salbutamol, tiotropium bromide, triamcinolone acetonide, cetirizine.

HES, hypereosinophilic syndrome; IS, immunosuppressive therapy; OCS, oral corticosteroids.

The most common medical conditions at screening varied across the HES duration subgroups: respiratory, thoracic and mediastinal disorders in the ≤5 years group (34%), and metabolism and nutrition disorders in the >5–≤10 and >10 years groups (45% and 50%, respectively) (**Table 1**). Most patients (92%) were receiving HES therapy at baseline. OCS was the most common baseline HES therapy across all disease duration groups, with most patients receiving >0–≤20 mg/day prednisone equivalent (**Table 1**).

### Characterization of flare symptoms

**Figure 1:**
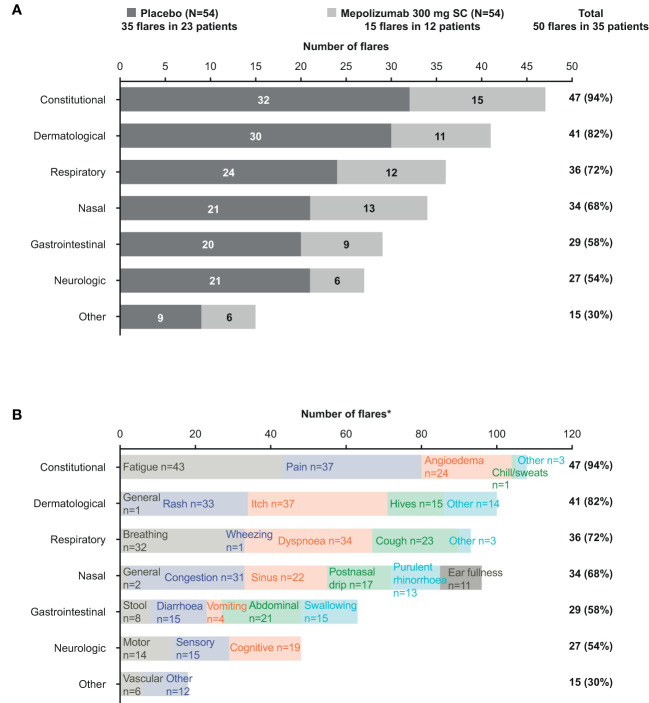
Frequency of flare symptoms by category and treatment group **(A)** and by category and term **(B)**. *Each flare could be categorized with more than one individual symptom and across several symptom categories. SC, subcutaneous.

In total, fewer patients receiving mepolizumab experienced flares than placebo; the total number of flare events reported was also lower with mepolizumab than placebo (15 vs 35, **Supplementary Table 2**). Overall, the most frequently reported flare symptoms were constitutional (47/50; 94%), dermatological (41/50; 82%) and respiratory (36/50; 72%) **(** **Figure 1A** **)**. Flares reported in patients receiving mepolizumab compared with placebo were generally similar in terms of the frequency of symptoms reported.

Individual flare symptom terms within each symptom category were described for the total population (**Figure 1B**). Overall, fatigue was the most common symptom reported, followed by pain and itch. By symptom category, fatigue and pain were the most common constitutional flare symptoms recorded; the most common dermatological flare symptoms were itch and rash, while breathing and dyspnea were the most common respiratory flare symptoms.

### Characterization of flare symptoms by subgroup

Across all baseline blood eosinophil count subgroups, fewer patients receiving mepolizumab experienced flares than those receiving placebo; a finding consistent with the total population (**Supplementary Table 2**). The total number of flare events reported was also lower with mepolizumab than placebo in all baseline blood eosinophil count subgroups (**Supplementary Table 2**).

**Figure 2:**
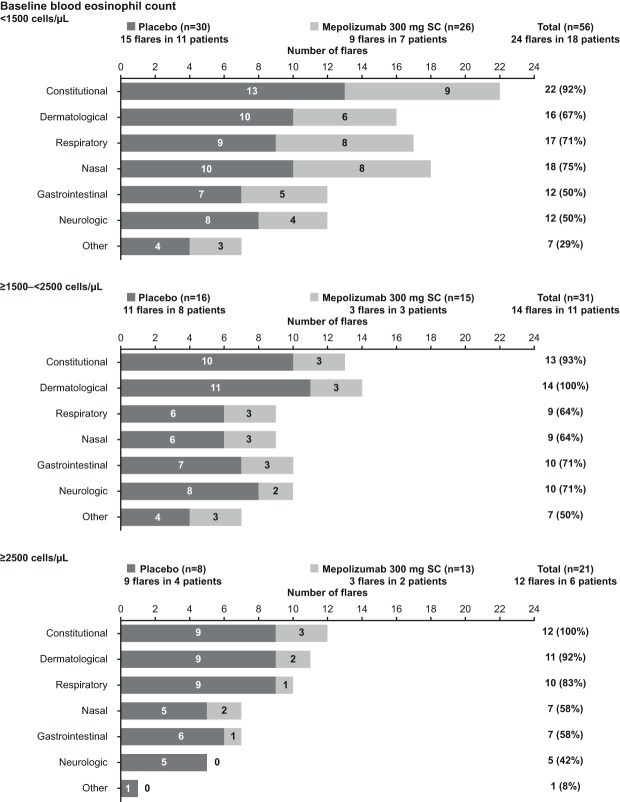
Frequency of flare symptoms by category and treatment group, stratified by baseline blood eosinophil count category. SC, subcutaneous.

The most frequently reported flare symptoms by category in all baseline blood eosinophil count subgroups (**Figure 2**) were similar to those reported in the total population (**Figure 1A**).

**Figure 3:**
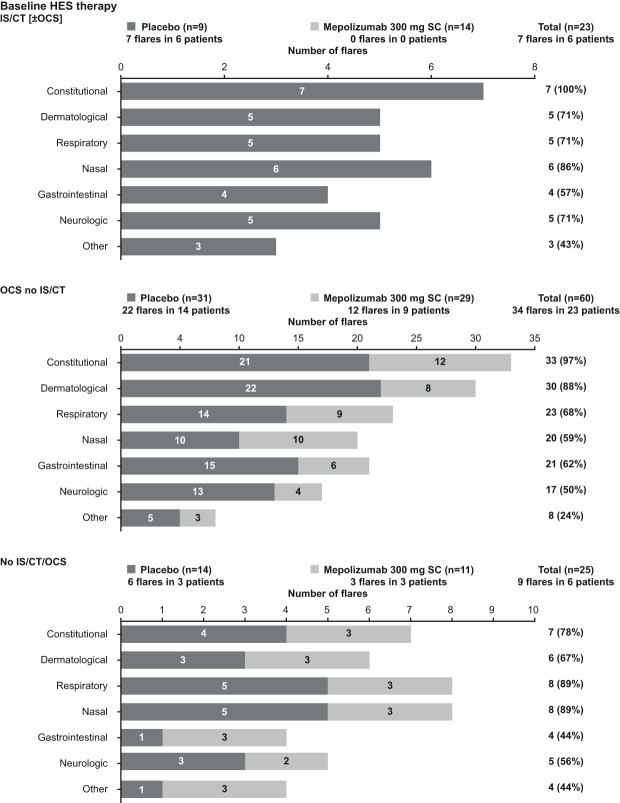
Frequency of flare symptoms by category and treatment group, stratified by baseline therapy (IS/CT [±OCS], OCS no IS/CT, No IS/CT/OCS). HES, hypereosinophilic syndrome; IS/CT, immunosuppressive/cytotoxic therapy; OCS, oral corticosteroids; SC, subcutaneous.

Across all baseline therapy subgroups, a lower proportion of patients receiving mepolizumab experienced flares than those receiving placebo; the total number of flares reported was also lower with mepolizumab than placebo (**Supplementary Table 2**). As in the baseline blood eosinophil count subgroups, the frequency of flare symptoms across all baseline HES therapy subgroups (**Figure 3**) followed a similar trend to the total population (**Figure 1A**).

**Figure 4:**
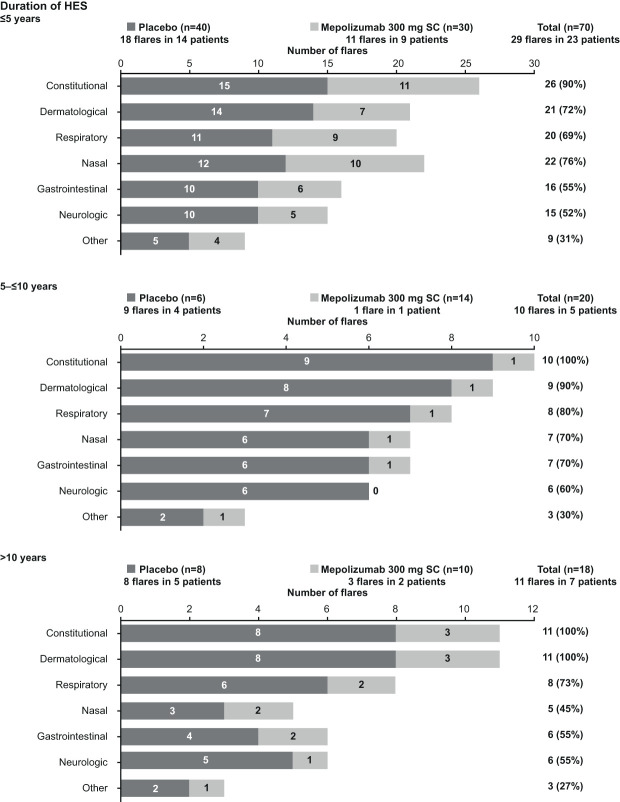
Frequency of flare symptoms by category and treatment group, stratified by duration of HES. HES, hypereosinophilic syndrome; SC, subcutaneous.

When patients were stratified by duration of HES, a lower proportion of patients receiving mepolizumab experienced flares than those receiving placebo, especially in patients with a disease duration >5 years; the total number of flares reported was also lower with mepolizumab than placebo in all subgroups and patients with the shortest disease duration (≤5 years) had the greatest number of flare events, irrespective of treatment arm (**Supplementary Table 2**). Consistent with the other subgroup analyses, the frequency of flare symptoms by category across the duration of HES subgroups was generally similar (**Figure 4**).

### Duration of flares

**Figure 5:**
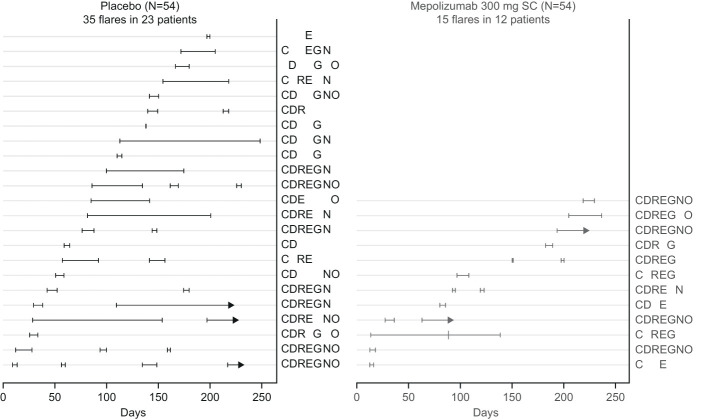
Flare duration and associated symptoms for each patient. Days are the study days since randomization (Day 0 is the study start). Each light line indicates an individual patient and each darker line indicates duration of a flare in patients receiving placebo (black) or mepolizumab (light grey). Two patients (one in each treatment group) experienced flares that overlapped with a previous flare (meeting either flare definition) by less than 14 days; these cases are shown on this figure as separate flares but counted as a single flare when summarizing the number and rate of HES flares. For those flares with missing end dates (arrows), the duration of flare was calculated up to date of study withdrawal. Codes indicate symptoms experienced during any flare. C, constitutional; D, dermatological; R, respiratory; E, nasal (ear, nose, throat); G, gastrointestinal, N, neurologic; O, other; SC, subcutaneous.

Over the study duration, mepolizumab treatment was associated with a lower adjusted annualized mean rate of flares meeting definition a) compared with placebo (0.44 vs 1.05;p=0.011). Furthermore, mepolizumab treatment was associated with a shorter median (range) duration of flares (10.0 [4, 126] days) compared with placebo (26.0 [1, 154] days). Patients receiving mepolizumab experienced fewer flares and a shorter duration of flares versus placebo, per patient; all patients, except two in the placebo and one in the mepolizumab treatment group, reported ≥3 symptoms during a flare (**Figure 5**).

## Discussion

Currently, descriptions of clinical manifestations of flares in large numbers of patients with HES are limited. Our *post hoc* analysis of data from the 200622 study, in which over 100 patients with HES were enrolled, highlighted that disease flares were associated with symptoms linked to multiple organ systems, including dermatological and respiratory symptoms. We also showed that the median duration of flares was reduced by at least half with mepolizumab compared with placebo. Together these data provide valuable insights into the clinical presentation of disease flares and the impact of mepolizumab in patients with HES.

We found that constitutional symptoms were the most frequent flare symptom, with nearly all patients reporting these events, which is consistent with an analysis of disease symptoms in 88 patients with HES (6). The results of our analysis also demonstrated that dermatological flare symptoms were reported in over 80% of flares and respiratory symptoms in over 70% of flares. Previous studies have shown that dermatological symptoms and respiratory symptoms are the most commonly reported HES manifestations (3, 5). Of the dermatological flare symptoms described in our analysis, itch and rash were the most common, and for respiratory flare symptoms, breathing and dyspnea were the most common. In a study by Kovacs et al. (7), which described general symptoms in 26 patients with HES, the constitutional symptoms itch and rash and the pulmonary symptom shortness of breath, were also reported in the greatest number of patients (7). Across all symptom categories, we identified fatigue as the most common symptom, a finding consistent with the Kovacs study (7), which showed fatigue as the most commonly reported HES symptom (65% of patients). The results from our analysis also showed that flare symptoms were similar irrespective of baseline blood eosinophil count, baseline HES therapy or duration of HES and, overall, our data further strengthen and quantify the most common flare symptoms experienced by patients with HES.

In the 200622 study, mepolizumab was shown to be associated with a 50% reduction in the proportion of patients experiencing at least one flare (meeting either definition a or b) and a 66% reduction in the annualized flare rate versus placebo (22). We explored the impact of mepolizumab on flares meeting protocol definition a) further in this analysis of 200622 study data and found that the median duration of flares was markedly reduced for patients receiving mepolizumab compared with those receiving placebo. This finding indicates that mepolizumab provides clinical benefit by reducing both the number and duration of disease flares. This is a clinically important finding as flares may persist for many months, which often results in patients receiving long-term treatment with OCS with or without IS/CT therapy. By reducing the occurrence of flares, mepolizumab treatment is therefore likely to allow patients to lower their exposure to OCS and subsequently reduce the substantial late-onset side effects associated with OCS use (1, 3, 13, 15).

In our analysis, the prevalence of comorbidities at baseline, including metabolism and nutrition disorders and endocrine disorders, was shown to increase with increasing HES disease duration. OCS have traditionally been the initial treatment of choice for patients with HES (9); however, long-term OCS use is associated with numerous side effects including bone fractures, cardiovascular disease, hyperglycemia and obesity (23). As such, the comorbidities reported in our analysis may reflect the long-term complications of OCS use highlighting the need for OCS-sparing therapies for patients with HES. Importantly, mepolizumab has previously been shown to have an OCS-sparing effect in patients with HES (5, 24). In a Phase III study (NCT00086658) assessing the efficacy of mepolizumab 750 mg given intravenously in patients with HES, 87% of patients receiving mepolizumab achieved a reduction in prednisone dose to ≤10 mg per day for more than 8 consecutive weeks versus 43% of those receiving placebo (5). In a 20-week open-label extension study (OLE; NCT03306043) continuing from the 200622 study, where all patients received mepolizumab 300 mg SC, nearly a third of patients who had received OCS treatment within the first 4 weeks of the OLE study were able to reduce their average daily OCS by at least 50% (24). Mepolizumab is therefore likely to provide benefits to patients not just in terms of disease flare reduction, but also by minimizing the use of other therapies that are associated with significant adverse events.

While the limitations of the 200622 study have been reported previously (22), the current analysis was further limited by its *post hoc* nature. Furthermore, flare symptom descriptions were limited by the extent of narrative provided by the investigator and by the total number of flares documented; the number of patients in each subgroup was also small. Moreover, patients with FIP1L1-PDGFRα-positive HES were excluded from the 200622 study, and HES subcategories, such as idiopathic, lymphocytic, and myeloproliferative HES (2, 8), were not identified. In addition, flare symptoms were identified following clinical review of flare narratives reported by the investigators and, as such, there may have been variability in their report and interpretation; flare symptoms reported by physicians may also differ to those reported by the patient (7). It is also of note that physicians were blinded to blood eosinophil counts during the study, which would normally be routinely used to diagnose a HES flare in the clinic. For the subgroup analyses, we relied on a blood eosinophil count reading at baseline only; however, it is well known that blood eosinophil counts vary over time and are influenced by previous treatments (25–27). Therefore, results relying on one blood eosinophil count reading at baseline should be interpreted with caution. Finally, we reported HES disease duration based on clinical diagnosis; however, because of the heterogenous clinical presentation of HES (1, 3–5), disease onset is likely to precede clinical diagnosis. As such, using the clinical duration of HES may be deemed somewhat arbitrary. Nonetheless, our study provides valuable insight into the nature of flares experienced by patients with HES.

In conclusion, patients with HES experience heterogeneous flare symptoms, irrespective of baseline blood eosinophil count, baseline therapy or clinical duration of disease. Together, the frequency of constitutional symptoms and the heterogenous nature of flare symptoms highlights the challenges faced by healthcare professionals in treating flares and measuring improvements in symptoms in patients with HES. Importantly, mepolizumab was associated with meaningful reductions in both the quantity and duration of flares versus placebo, providing further evidence of the clinical benefits of mepolizumab in patients with HES.

## Participating investigators of the HES mepolizumab study group

Participating investigators: Gabriel Ricardo García, Adriana Sosso, Luis Wehbe, Anahí Yañez; Daniël Blockmans, Florence Roufosse, Martti Anton Antila, Daniela Blanco, Sergio Grava, Marina Andrade Lima, Andreia Luisa Francisco Pez, Stanislas Faguer, Mohamed A. Hamidou, Jean-Emmanuel Kahn, Guillaume Lefévre, Knut Brockow, Peter M. Kern, Juliana Schwabb, Bastian Walz, Tobias Welte, Fabrizio Pane, Alessandro M. Vannucchi, Ruth Cerino-Javier, Alfredo Gazca-Aguilar, Dante D. Hernández-Colín, Héctor Glenn Valdéz-López, Izabela R. Kupryś-Lipińska, Jacek Musial, Witold Prejzner, Eniko Mihaly, Viola Popov, Mihnea Tudor Zdrenghea, Sergey V. Gritsaev, Vladimir Ivanov, Nikolay Tsyba, Aránzazu Alonso, Georgina Espígol-Frigolé, Maria Laura Fox, Regina Garcia Delgado, Jesús María Hernández Rivas, Guillermo Sanz Santillana, Ana Isabel González, Andrew J. Wardlaw, Praveen Akuthota, Joseph H. Butterfield, Geoffrey L. Chupp, John B. Cox, Gerald J. Gleich, Devi Jhaveri, Marc E. Rothenberg

## Affiliation details of participating investigators in the HES mepolizumab study group

**Argentina**: Gabriel Ricardo García, Centro Platense en Investigaciones Respiratorias, Buenos Aires; Adriana Sosso, Centro de Investigaciones Clinicas, Buenos Aires; Luis Wehbe, Instituto Ave Pulmo-Fundacion Enfisema, Buenos Aires; Anahí Yañez, Investigaciones en Alergia Enfermedades Respiratorias-Consultorios Medicos, Buenos Aires; **Belgium**: Daniël Blockmans, UZ Leuven – Campus Gasthuisberg, Leuven; Florence Roufosse, Hôpital Erasme, Brussels; **Brazil**: Martti Anton Antila, Clinica de Alergia Martti Antila and Hospital Santa Lucinda, Sorocaba, São Paulo; Daniela Blanco, Hospital São Lucas da PUCRS, Centro de Pesquisa Clinica, Porto Alegre, Rio Grande Do Sul; Sergio Grava, Parana Medical Research Center, Maringá, Paraná; Marina Andrade Lima, Hospital Dia do Pulmão, Departamento de Pesquisa Clinica, Blumenau, Santa Catarina; Andreia Luisa Francisco Pez, Pesquisare Saúde Sociedade Simples Ltda, Santo André-SP, São Paulo; **France**: Stanislas Faguer, Centre Hospitalier Universitaire de Toulouse - Hôpital Rangueil, Toulouse; Mohamed A. Hamidou, Centre Hospitalier Universitaire de Nantes, Nantes; Jean-Emmanuel Kahn, Hôpital Foch, Service de Médecine Interne, Suresnes; Guillaume Lefévre, Centre Hospitalier Régional Universitaire de Lille – Hôpital Claude Huriez, Lille; **Germany**: Knut Brockow, Technische Universitaet Muenchen, Klinik and Poliklinik für Dermatologie, Muenchen; Peter M. Kern, Klinikum Fulda-MVZ Medizinische Klinik IV, Fulda; Juliana Schwaab, University Hospital Mannheim, Heidelberg University, Mannheim, **Germany**; Bastian Walz, Kreiskliniken Esslingen Klinik Kirchheim, Kirchheim unter Teck; Tobias Welte, Medizinische Hochschule, Hannover; **Italy**: Fabrizio Pane, Azienda Ospedaliera Universitaria Federico II, U. O. Ematologia e Trapianti di Midollo, Napoli; Alessandro M. Vannucchi, Azienda Ospedaliero-Universitaria di Careggi, Dipartimento di Medicina Sperimentale e Clinica, Firenze; **Mexico**: Ruth Cerino-Javier, Hospital Angeles de Villahermosa, Villahermosa, Tabasco; Dante D. Hernández-Colín, Instituto Jalisciense de Investigación Clinica, Sociedad Anónima de Capital Variable, Guadalajara, Jalisco; Héctor Glenn Valdéz-López, CRI Centro Regiomontano de Investigacion SC, Monterrey, Nuevo León; **Poland**: Izabela R. Kupryś-Lipińska, Samodzienly Publiczny Zaklad Opieki Zdrowotnej, Uniwersytecki Szpital Kliniczny nr 1 im. Norberta Barlickiego UM W Łodzi, Łódź, Łódźkie; Jacek Musial, Szpital Uniwersytecki w Krakowie, Oddzial Kliniczny Alergii i Immunologii, Krakow; Witold Prejzner, Uniwersyteckie Centrum Kliniczne, Klinika Hematologii i Transplantologii, Gdansk; **Romania**: Eniko Mihaly, SC Dora Medicals SRL Targu Mures; Viola Popov, Centrul Medical Unirea SRL – Policlinica Enescu, Bucharest; Mihnea Tudor Zdrenghea, Institutul Oncologic “Prof Dr Ion Chiricuta”, Cluj-Napoca; **Russia**: Sergey V. Gritsaev, Russian Hematology and Transfusiology Research Center, St Petersburg; Vladimir Ivanov, Almazov National Medical Research Center, Ministry of Health of Russian Federation, St Petersburg; Nikolay Tsyba, Scientific Advisory Department of Chemotherapy of Myeloproliferative Disorders, Hematology Research Center, Moscow; **Spain**: Aránzazu Alonso, Hospital Quirón Madrid Servicio de Hematologia, Madrid; Georgina Espígol-Frigolé, Hospital Clinic i Provincial de Barcelona, Barcelona; Maria Laura Fox, Hospital Vall d Hebrón, Barcelona; Regina Garcia Delgado, Hospital Virgen de la Victoria, Campus Universitario de Teatinos, Málaga; Jesús María Hernández Rivas, Hospital Universitario de Salamanca, 1°C Planta/Laborotoria de Hematología, Salamanca; Guillermo Sanz Santillana, Hospital Universitari i Politecnic La Fe, Valencia; Ana Isabel González, Hospital Universitari i Politecnic La Fe, Valencia; **United Kingdom**: Andrew J. Wardlaw, Institute for Lung Health, Department of Allergy and Respiratory Medicine, Glenfield Hospital, Respiratory Biomedical Research Unit, Leicester; **United States**: Praveen Akuthota, University of California, San Diego, California; Joseph H. Butterfield, Mayo Clinic, Rochester, Minnesota; Geoffrey L. Chupp, Yale New Haven Hospital, New Haven, Connecticut; John B. Cox, Medical University of South Carolina Investigational Drug Services, Charleston, South Carolina; Gerald J. Gleich, University of Utah, Dermatology Midvalley Health Center, Murray, Utah; Devi Jhaveri, Ohio Clinical Research Associates, Mayfield Heights, Ohio; Marc E. Rothenberg, Cincinnati Children’s Hospital Medical Center, Cincinnati, Ohio.

## List of approving ethics committees for the HES 200622 study

The local institutional review board or ethics committee at each study center oversaw trial conduct and documentation. The approving ethics committees were as follows:

**Argentina**: Instituto Medico Platense, Boulevard 51 Nro 315, La Plata, Buenos Aires, B1900AVG; Comité de Ética en Investigación INAER, Arenales 3146 1°A, Ciudad Autonoma de Buenos Aires, Buenos Aires, C1425BEN; Comite de Etica en Investigacion, Instituto Ave Pulmo, Carlos M. Alvear 3345, Mar del Plata, Buenos Aires, B7602DCK; **Belgium**: Hôpital Erasme, Route de Lennik 808, Brussels, 1070; **Brazi**l: Comite de Etica em Pesquisa do Investiga- Instituto de Pesquisas, Avenue Romeu Tortima, 739 - CEP: 13084-791, Campinas, São Paulo, 13084-791; Hospital Sao Lucas da PUCRS, Avenue Ipiranga, 6690, Porto Alegre, Rio Grande Do Sul, 90610-000; Comitê de Ética em Pesquisa - CESUMAR, Avenue Guedner, 1610 - Bloco 10 - Jardim Aclimação, Maringa, Paraná, 87050-900; CEPH - FURB, Bloco A, 2° Andar, Rua Antônio da Veiga, 140, Blumenau, Santa Catarina, 89.012-900; CEP Centro Universitario Saude ABC, Avenida Principe de Gales 821, Santo Andre, São Paulo, 9060650; **France**: Comité de Protection des Personnes Ile de France III - Hôpital Tarnier, 89, rue d’Assas, Paris, 75006; **Germany**: Ethikkommission der Medizinischen Hochschule Hannover, Carl-Neuberg-Strasse 1, Hannover, Niedersachsen, 30625; **Italy**: Comitato Etico Università Federico II di Napoli, Segreteria Tecnico-Amministrativa, Edificio 20, Via Pansini, 5, Napoli, Campania, 80131; Comitato Etico Reg. Toscano “Area Vasta Centro”, Segreteria Scientifico-Amministrativa - Nuovo Ingresso Careggi, pad. 3 - Didattica, Largo Brambilla, 3, Firenze, Toscana, 50134; **Mexico**: Instituto Jalisciense de Investigación Clínica, Sociedad Anónima de Capital Variable, Penitenciaria 20, Guadalajara, Jalisco, 44100; Biomedical Research G And L, Sociedad De Responsabilidad Limitada De Capital Variable, Avenida La Calma 3475 Colonia La Calma, Zapopan, Jalisco, 45070; CEIIC Comite de Etica en Independiente en Investigacion Cientifica, Aguilar Sur 669 Colonia Obispado CP, Monterrey, Nuevo León, 64060; **Poland**: Komisja Bioetyczna Uniwersytetu Jagiellonskiego, Grzegorzecka 20, Krakow, 31-531; **Romania**: Comisia Nationala de Bioetica a medicamentelor si a Dispozitivelor Medicale, Pavilion K, Spitalul Clinic Colentina, Sos. Stefan cel Mare nr. 19-21, Bucuresti, 20125; **Russia**: Russian Hematology and Transfusiology Research Center, 16, 2nd Sovetskaya strasse, Saint Petersburg, 191024; Almazov National Medical Research Center, 2, Akkuratova street, Saint Petersburg, 197341; Hematology Research Center, 4A, Novyi Zykovskyi proezd, Moscow, 125167; **Spain**: Hospital la Paz, Paseo de la Castellana, 261, Madrid, 28046; **United Kingdom**: East Midlands Nottingham 2, The Old Chapel, Royal Standard Place, Nottingham, NG1 6FS; **United States**: Human Research Protection Program, University of California San Diego, 9500 Gilman Drive, LaJolla, California, 92037; Mayo Institutional Review Board, Mayo Clinic, 200 First Street, South West, Rochester, Minnesota, 55905; Yale University Human Investigation Committee, 25 Science park– 3^rd^ Floor, 150 Munson Street, New Haven, Connecticut, 06520; Western Institutional Review Board, 1019 39th Avenue South East, Suite 120, Puyallup, Washington, 98374; University of Utah, Institutional Review Board, Research Administration Building, 75 South 2000 East, Salt Lake City, Utah, 84112; Copernicus Group, Suite 200, 5000 Centre Green Way, Cary, North Carolina, 27513; Cincinnati Children’s Hospital Medical Center Institutional Review Board, 3333 Burnet Avenue, Location R 5392, Cincinnati, Ohio, 45229.

## Data availability statement

Anonymized individual participant data and study documents for the parent study can be requested for further research from www.clinicalstudydatarequest.com.

## Ethics statement

The results presented are from a multi-center study. Details of all approving ethics committees are given at the end of the main article text. The patients/participants provided their written informed consent to participate in this study.

## Author contributions

JB, SWY, and JS were involved in the conception or design of the study. GL and FP were involved in the acquisition of the data. All authors contributed to the analysis or interpretation of data, drafted the work, or revised it critically for important intellectual content, gave final approval of the version to be published, and agreed to be accountable for all aspects of the work.

## Acknowledgments

Editorial support (in the form of writing assistance, including preparation of the draft manuscript under the direction and guidance of the authors, collating and incorporating authors’ comments for each draft, assembling tables and figures, grammatical editing and referencing) was provided by Katie Crossland, PhD, at Fishawack Indicia Ltd, UK, part of Fishawack Health.

## Conflict of interest

This study was funded by GSK (GSK ID: 200622; NCT02836496). The study sponsor had a role in study design, data collection, data analysis and interpretation, and in the writing of the study report. The sponsor did not place any restrictions on access to data or statements made in the manuscript report. Authors had full access to all study data and had final responsibility to submit the manuscript for publication. FP received advisory and speaker fees from Novartis, Bristol Myers Squibb, ARIAD Pharmaceuticals, GSK, AMGEN, Janssen, AbbVie, and Jazz Pharmaceutical, and research grants from Novartis and ARIAD Pharmaceuticals. GL received advisory fees from Shire, Takeda, AstraZeneca, and Sanofi Genzyme and has received research grants and travel/ accommodation expenses from AstraZeneca, Shire, Octapharma, and GSK. JB is an employee of GSK and own stocks/shares. NK, SWY and JS are former employees of GSK and own stocks/shares.

## Publisher’s note

All claims expressed in this article are solely those of the authors and do not necessarily represent those of their affiliated organizations, or those of the publisher, the editors and the reviewers. Any product that may be evaluated in this article, or claim that may be made by its manufacturer, is not guaranteed or endorsed by the publisher.
