# Supplementary material for: Characterization of disease flares and impact of mepolizumab in patients with hypereosinophilic syndrome
Source: Front Immunol. 2022 Aug 26;13:935996. doi: 10.3389/fimmu.2022.935996 (PMC9462399; doi:10.3389/fimmu.2022.935996)
Supplement: Supplementary file 1 [file DataSheet_1.docx]

**Supplementary File 1**: **HES Core Assessments form**

In the 200622 study, patients with HES were evaluated by the investigator using the HES Core Assessments form as a guide at each clinic visit. The HES Core Assessments form consisted of clinical signs and symptoms that reflected the heterogenous nature of HES observed in clinical practice. Throughout the study, the investigator determined whether worsening of signs/symptoms supported an increase in HES therapy, and also prepared a narrative for each HES flare. Before diagnosing a HES flare, other possible causes for the change in clinical symptoms were ruled out. Findings of the HES Core Assessments form were recorded in an electronic device.
