# Supplementary material for: Characterization of disease flares and impact of mepolizumab in patients with hypereosinophilic syndrome
Source: Front Immunol. 2022 Aug 26;13:935996. doi: 10.3389/fimmu.2022.935996 (PMC9462399; doi:10.3389/fimmu.2022.935996)
Supplement: Supplementary file 2 [file Table_1.docx]

**Supplementary Table 1** HES Core Assessments form

| **Symptoms** | **Assessment** |
| --- | --- |
| **Constitutional**  Fatigue  Pain (including but not limited to muscle, joint, general pain)  Angioedema (swelling under the skin) | Each symptom rated using a 0–3 scale |
| **Dermatologic**  Rash  Itch  Hives  Others (specify) | Each symptom rated using a 0–3 scale |
| **Gastrointestinal**  Average number of vomiting a day in the past week  Average number of diarrhea a day in the past week  Average number of stools a day in the past week  Abdominal pain  Difficulty in swallowing food | Each symptom rated using a 0–3 scale |
| **Respiratory**  Breathing symptoms such as shortness of breath and wheezing  Dyspnea (shortness of breath)  Cough | Each symptom rated using a 0–3 scale |
| **Nasal (ear, nose, throat)**  Nasal congestion  Sinus headache/facial pain/pressure  Postnasal drip (drainage down the back of the throat)  Purulent rhinorrhea (discolored & thick nasal discharge)  Ear fullness | Each symptom rated using a 0–3 scale |
| **Cardiovascular***  Heart failure classification for functional capacity  Heart failure classification for objective assessment | Classes I-IV  Classes A-D |
| **Neurologic**  Sensory  Motor  Cognitive and mental status change | Each symptom rated using a 0–3 scale |
| **Others**  Vascular, venous, arterial, loss of pulse, splinter hemorrhage, renal failure, splenomegaly, other (specify) | Each identified symptom rated using a 0–3 scale |

0–3 scale symptom score: 0 for not present or no impact; 1 for present but minimal impact; 2 for significant impact on daily activities; 3 for incapacitating. *American Heart Association: classes of heart failure:^26^ Class I: No limitation of physical activity. Ordinary physical activity does not cause undue fatigue, palpitation or dyspnea; Class II: Slight limitation of physical activity. Comfortable at rest. Ordinary physical activity results in fatigue, palpitation or dyspnea; Class III: Marked limitation of physical activity. Comfortable at rest. Less than ordinary activity causes fatigue, palpitation or dyspnea; Class IV: Unable to carry on any physical activity without discomfort. Symptoms of heart failure at rest. If any physical activity is undertaken, discomfort is increased. Class A: No objective evidence of cardiovascular disease; Class B: Objective evidence of minimal cardiovascular disease; Class C: Objective evidence of moderately severe cardiovascular disease; Class D: Objective evidence of severe cardiovascular disease. HES, hypereosinophilic syndrome.
