# Supplementary material for: Characterization of disease flares and impact of mepolizumab in patients with hypereosinophilic syndrome
Source: Front Immunol. 2022 Aug 26;13:935996. doi: 10.3389/fimmu.2022.935996 (PMC9462399; doi:10.3389/fimmu.2022.935996)
Supplement: Supplementary file 3 [file Table_2.docx]

## **Supplementary Table 2** Proportion of patients with flares and frequency of flares* by analysis population

| **Patient population** | **Total** | | | **Placebo** | | | **Mepolizumab 300 mg SC** | | |
| --- | --- | --- | --- | --- | --- | --- | --- | --- | --- |
|  | **Total patients** | **Patients with flares, n (%)** | **Flare events, n** | **Patients in group** | **Patients with flares, n (%)** | **Flare events, n** | **Patients in group** | **Patients with flares, n (%)** | **Flare events, n** |
| **ITT** | N=108 | 35 (32) | 50 | N=54 | 23 (43) | 35 | N=54 | 12 (22) | 15 |
| **Baseline blood eosinophil count, cells/µL** | | | |  | | |  | | |
| <1500 | n=56 | 18 (32) | 24 | n=30 | 11 (37) | 15 | n=26 | 7 (27) | 9 |
| ≥1500–<2500 | n=31 | 11 (35) | 14 | n=16 | 8 (50) | 11 | n=15 | 3 (20) | 3 |
| ≥2500 | n=21 | 6 (29) | 12 | n=8 | 4 (50) | 9 | n=13 | 2 (15) | 3 |
| **Baseline therapy** | | | |  | | |  | | |
| IS/CT [±OCS] | n=23 | 6 (26) | 7 | n=9 | 6 (67) | 7 | n=14 | 0 (0) | 0 |
| OCS no IS/CT | n=60 | 23 (38) | 34 | n=31 | 14 (45) | 22 | n=29 | 9 (31) | 12 |
| No IS/CT/OCS | n=25 | 6 (24) | 9 | n=14 | 3 (21) | 6 | n=11 | 3 (27) | 3 |
| **Duration of HES, year** | | | |  | | |  | | |
| ≤5 | n=70 | 23 (33) | 29 | n=40 | 14 (35) | 18 | n=30 | 9 (30) | 11 |
| 5–≤10 | n=20 | 5 (25) | 10 | n=6 | 4 (67) | 9 | n=14 | 1 (7) | 1 |
| >10 | n=18 | 7 (39) | 11 | n=8 | 5 (63) | 8 | n=10 | 2 (20) | 3 |

*Only flares meeting protocol definition a), i.e., a HES-related clinical manifestation based on a physician-documented change in clinical signs or symptom that required either an increased dose of maintenance OCS ≥10 mg prednisone equivalent/day for 5 days or an increase in/addition of IS/CT, are included. HES, hypereosinophilic syndrome; IS/CT, immunosuppressive/cytotoxic; ITT, intent-to-treat; OCS, oral corticosteroid; SC, subcutaneous.
